# Latin America validation of FACED score in patients with bronchiectasis: an analysis of six cohorts

**DOI:** 10.1186/s12890-017-0417-3

**Published:** 2017-04-26

**Authors:** Rodrigo Athanazio, Mônica Corso Pereira, Georgina Gramblicka, Fernando Cavalcanti-Lundgren, Mara Fernandes de Figueiredo, Francisco Arancibia, Samia Rached, David de la Rosa, Luis Máiz-Carro, Rosa Girón, Casilda Olveira, Concepción Prados, Miguel Angel Martinez-Garcia

**Affiliations:** 1 Pulmonary Division, Heart Institute (InCor) do Hospital das Clinicas da Faculdade de Medicina da Universidade de São Paulo, Av Dr Eneas de Carvalho Aguiar, 44 – 5 andar (Pneumologia), São Paulo, 05403-900 Brazil; 2 Pneumology Service, State University of Campinas (Unicamp), São Paulo, Brazil; 3 Pneumology Service, Hospital del Tórax. Dr A. Cetrángolo, Buenos Aires, Argentina; 4 Pneumology Service, Hospital Octávio de Freitas, Recife, Brazil; 5 Pneumology Service, Hospital de Messejana, Fortaleza, Brazil; 6 Pneumology Service, Instituto Nacional del Tórax, Santiago de Chile, Chile; 7 Pneumology Unit, Hospital Platón, Barcelona, Spain; 8 Pneumology Service, Hospital Universitario Ramón y Cajal, Madrid, Spain; 9 Pneumology Service, Hospital Universtario La Princesa, Madrid, Spain; 10 Pneumology Service, Hospital General de Málaga, Málaga, Spain; 11 Pneumology Service, Hopital Universitario La Paz-Carlos III, Madrid, Spain; 12 Pneumology Service, Polytechnic and University La Fe Hospital, Valencia, Spain

**Keywords:** Bronchiectasis, Prognosis, Validation studies, FACED score, Mortality

## Abstract

**Background:**

The FACED score is an easy-to-use multidimensional grading system that has demonstrated an excellent prognostic value for mortality in patients with bronchiectasis. A Spanish group developed the score but no multicenter international validation has yet been published.

**Methods:**

Retrospective and multicenter study conducted in six historical cohorts of patients from Latin America including 651 patients with bronchiectasis. Clinical, microbiological, functional, and radiological variables were collected, following the same criteria used in the original FACED score study. The vital status of all patients was determined in the fifth year of follow-up. The area under ROC curve (AUC-ROC) was used to calculate the predictive power of the FACED score for all-cause and respiratory deaths and both number and severity of exacerbations. The discriminatory power to divide patients into three groups of increasing severity was also analyzed.

**Results:**

Mean (SD) age of 48.2 (16), 32.9% of males. The mean FACED score was 2.35 (1.68). During the follow up, 95 patients (14.6%) died (66% from respiratory causes). The AUC ROC to predict all-cause and respiratory mortality were 0.81 (95% CI: 0.77 to 0.85) 0.84 (95% CI: 0.80 to 0.88) respectively, and 0.82 (95% CI: 078–0.87) for at least one hospitalization per year. The division into three score groups separated bronchiectasis into distinct mortality groups (mild: 3.7%; moderate: 20.7% and severe: 48.5% mortality; *p* < 0.001).

**Conclusions:**

The FACED score was confirmed as an excellent predictor of all-cause and respiratory mortality and severe exacerbations, as well as having excellent discriminative capacity for different degrees of severity in various bronchiectasis populations.

**Electronic supplementary material:**

The online version of this article (doi:10.1186/s12890-017-0417-3) contains supplementary material, which is available to authorized users.

## Background

The multidimensional nature of bronchiectasis means that any analysis of a single variable is insufficient to assess its severity or prognosis [1]. Accordingly, two multidimensional scales have been published in recent years, using several different variables that are easily obtained and have a proven capacity for an accurate prognosis of mortality: the FACED score [2] and the Bronchiectasis Severity Index (BSI) [3]. Both scores have been use to assess the severity of bronchiectasis in several publications [4–8].

The FACED score is a simple score that consists of five dichotomized variables: age, clinical aspects (dyspnea), lung function (FEV_1_), microbiology (chronic bronchial infection by *Pseudomonas aeruginosa*), and radiological findings (number of affected lung lobes in computed tomography). This score was developed by a Spanish group in 398 patients and showed excellent internal validity in 411 additional patients. It has demonstrated an excellent power to predict mortality (both all-cause and respiratory) within 5 years of diagnosis [2]. Its longer-term prognostic capacity (up to 15 years) has been confirmed by other series of European patients [9, 10].

However, before a new score can be fully accepted and reliably applied to clinical practice, its validity must be tested in different settings on new data from an appropriately assembled sample of subjects. This process is called external validation. It is further desirable that the sample chosen for external validation is selected with the same criteria as the initial series, while including patients whose characteristics are sufficiently different to enable an evaluation of the score’s breadth of application [11].

Therefore, the primary objective of this study was to perform an external validation of the FACED score for both overall and respiratory mortality in a large group of patients outside Europe from six historical cohorts of several countries in Latin America. The secondary objective was to evaluate the FACED score’s ability to predict exacerbations and hospitalizations.

## Methods

### Latin America validation sample

This is a retrospective and multicenter study of historical cohorts that included 672 patients from six cohorts from three different countries from Latin America (Argentina, Brazil, and Chile) with a diagnosis of bronchiectasis confirmed by high resolution computed tomography scan (HRCT scan). All data were collected from specialized bronchiectasis clinics and from patients that had at least 5 years of follow-up after the date of the radiological diagnosis of bronchiectasis. Patients were included starting from January 1, 2005. The inclusion and exclusion criteria were the same as those in the original study that constructed the FACED score, as previously published [2]. Data were also collected in all centers according to the same standardized protocol used in the FACED internal validation. The study was approved by the ethics committees of all the participating centers (Additional file 1: e-Appendix).

### Description of the FACED score

**Fig. 1:**
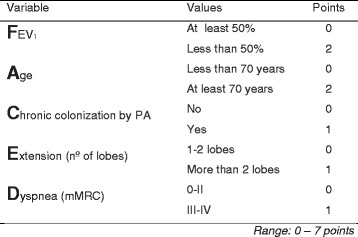
FACED score including cut-off points of the dichotomized variables and scoring of each variable. FEV_1_: forced expiratory volume in the first second, mMRC: modified medical research council; PA: *Pseudomonas aeruginosa*

The FACED score consists of five dichotomized variables: FEV_1_, age, presence of chronic colonization by *Pseudomonas aeruginosa*, dyspnea measured by the modified MRC dyspnea scale (mMRC) and number of pulmonary lobes affected on computed tomography (CT). The possible range of points is 0 to 7, with a higher score indicating greater severity of the disease. The division of the FACED score into three groups makes it possible to define bronchiectasis as mild (0–2 points), moderate (3–4 points), or severe (5–7 points) (Fig. 1).

### Variables

As in the study for the construction and internal validation of FACED score, all variables were collected in a standardized manner: general characteristics (age and gender); anthropometric (body mass index [BMI]); smoking habit; symptoms (dyspnea mMRC, appearance of sputum [mucous, mucopurulent, purulent]); presence of chronic respiratory failure (room air oxygen saturation < 90%); radiological findings; etiology of bronchiectasis; colonization by potentially pathogenic microorganisms (PPM), the number of exacerbations and hospitalizations in the year prior to inclusion in the study and during the follow-up; functional variables; and treatments. According to the original FACED paper, chronic colonization was defined as isolation of the same PPM after the diagnosis of bronchiectasis in three consecutive respiratory samples taken at least one month apart within a period of six months. In view of the difference between the care system of patients in Latin America, a less restrictive definition of chronic colonization was used such as at least two isolates of a PPM 3 months apart over 1 year [12]. All the variables were collected as close as possible to the date of the diagnosis of bronchiectasis to avoid interference from different treatments.

### Follow-up and end-points

The primary end-points of the study were all-cause and respiratory mortality after 5 years of follow-up from the diagnosis of bronchiectasis. The date and cause of death were confirmed by checking the centers’ digital databases and the relevant death certificates. The numbers of exacerbations and hospitalizations were also recorded during the 5 years of follow-up and these were presented as the ratio of events per year of follow-up.

### Statistical analysis

The statistical analysis was the same as that used for the construction and internal validation of the FACED score [2]. Data for the quantitative variables were tabulated as mean ± SD, while the qualitative were tabulated as the percentage of the total subjects. The chi-squared test was used for comparison of qualitative and dichotomic variables. The normality of the variables was confirmed with the Kolgomorov-Smirnov test.

Less than 10% of patients had missing data and were excluded from the analysis. The baseline characteristics of these patients were not different compared with the included ones.

Data from the different centers were compared with one-way ANOVA test. The diagnostic capacity to predict all-cause and respiratory mortality and also exacerbation/hospitalization rates of the FACED score was determined by tracing their corresponding area under ROC curves (AUC-ROC) and 95% confidence interval (CI). The greater the AUC-ROC, the better the predictive value of the FACED score. An AUC-ROC greater than 0.80 was established as excellent [13]. As in the original paper, FACED score was divided into 3 groups (tertiles) with progressively increasing severity (mild, moderate and severe), and each group’s capacity to predict mortality was calculated using Kaplan-Meier method. The two-by-two comparison between the different Kaplan-Meier curves was performed with the log-rank test. Significant difference was considered when *p* < 0.05.

## Results

Data were collected from 672 initial patients but 21 individuals were excluded (3 were under 18 years old and 18 did not present all the required variables). Finally, 651 patients were included in the analysis, and 95 of them (14.6%) died during the 5 years of follow-up. The main cause of death was related to respiratory complications (66% of cases). The general characteristics of these patients are presented in Table 1. The mean ± SD age of the overall sample was 48.2 ± 16.0 years, while 32.9% of the patients were male and 39.8% were chronically colonized by *Pseudomonas aeruginosa*. The most frequent known etiology of bronchiectasis was post-infective (including post-tuberculosis) in 40.3% of cases, followed by ciliary dyskinesia (9.0%). Thirty-one percent were of unknown etiology.

**Table 1:** Baseline characteristics of the international validation cohort

Variables	All patients (*n* = 651)
Age, years	48.2 ± 16.0
Gender, % of men	32.9%
Body-mass index, kg/m^2^	22.4 ± 11.5
Dyspnea (mMRC)	1.52 ± 1.0
Smoking (packs-year)	4.81 ± 12.8
Sputum appearance, %	
No sputum	18,6%
Mucous	27.2%
Mucopurulent	35.2%
Purulent	19.0%
Respiratory insufficiency, %	16.9%
Number of affected lobes	3.3 ± 1.5
Aetiology %	
Post-infective	40.3%
Idiopathic	31.3%
Primary ciliary diskinesia	9.0%
Airway disease^a^	5.1%
Rheumatologic disease	4.3%
Other causes	10.0%
FEV_1_, % predicted	54.7 ± 22.1
FVC, % predicted	67.2 ± 20.3
Chronic colonization, %	
*Pseudomonas aeruginosa*	39.8%
Exacerbations (previous year)	1.12 ± 1.4
Hospitalizations (previous year)	0.4 ± 0.8
Chronic treatment, %	
Systemic antibiotics	7.2%
Inhaled antibiotics	30.5%
Macrolides	17.3%
Oral corticoids	3.8%
Comorbidities, %	
Asthma	10.0%
Systemic arterial hypertension	9.8%
Pulmonary hypertension	4.0%
COPD	3.7%
Diabetes mellitus	3.4%
Chronic cardiac disease	3.2%
Death, %	14.6%

^a^COPD, asthma and bronchiolitis as the underlying disease that led to bronchiectasis

Table 2 presents the patients’ characteristics according to the six participating centers. Significant differences were observed in relation to their general, etiological, clinical, functional, radiological, and microbiological variables. Mortality rate ranged from 10.1% to 19.4% between centers. Table 2 also presents information about the original Spanish cohort for the FACED score, emphasizing several differences between the initial and international validation cohorts.

**Table 2:** Characteristics of patients of international validation cohort by participating center and the initial Spanish cohort [2]

	Argentina	Brazil 1	Brazil 2	Brazil 3	Brazil 4	Chile	*p*-value*	Initial Spanish cohort [2]
Subjects, (n)	108	83	101	103	187	69		819
Age in years	44.4 ± 16.8	45.1 ± 15.7	52.5 ± 17.7	47.1 ± 15.6	47.4 ± 14.5	54.5 ± 13.8	0.0001	58.7 ± 17.6
Male (%)	33.3%	25.3%	18.8%	42.7%	34.2%	43.5%	0.002	43.5%
Etiology								
Post-infective	55.6%	47.0%	60.4%	16.5%	24.6%	62.7%	0.00000	31.3%
Idiopathic	34.3%	25.3%	26.7%	44.7%	34.2%	5.9%	1	37.9%
Dyspnea mMRC score	1.16 ± 0.8	1.32 ± 1.3	1.90 ± 1.0	1.71 ± 0.9	1.53 ± 0.9	1.47 ± 1.0	0.00001	1.53 ± 1.1
Mucopurulent or purulent sputum appearance (%)	61.1%	75.9%	71.1%	52.4%	44.2%	34.8%	0.0001	35.4%
Respiratory insufficiency (%)	14.8%	13.3%	15.8%	29.1%	16.1%	10.1%	0.013	10.1%
Lobes affected	3.0 ± 1.3	2.8 ± 1.0	3.1 ± 1.2	3.7 ± 1.5	3.9 ± 1.6	2.6 ± 1.2	0.0001	2.52 ± 1.2
FEV1 % of predicted	62.2 ± 21.7	57.5 ± 21.4	58.4 ± 23.8	49.1 ± 21.5	49.6 ± 19.9	54.7 ± 22.0	0.000001	68.9 ± 25.9
Chronic colonization by *PA* (%)	58.3%	36.1%	23.8%	52.4%	40.6%	17.4%	0.0001	31.8%
Exacerbations in previous year**	1.04 (0.83)	2.6 (1.01)	1.25 (1.3)	0.81 (1.2)	0.7 (1.7)	0.9 (0.94)	0.001	2.52 (2.2)
Hospitalizations in previous year	0.47 ± 0.6	0.61 ± 0.8	0.28 ± 0.6	0.26 ± 0.6	0.28 ± 0.9	0.49 ± 0.6	0.005	0.7 ± 1.2
Exacerbation rate (per year)**	1.05 (0.6)	0.8 (0.6)	1.2 (0.92)	1.24 (1)	0.75 (0.97)	0.8 (0.58)	0.01	--
Hospitalization rate (per year)	0.4 (0.41)	0.3 (0.5)	0.42 (0.64)	0.37 (0.78)	0.12 (0.37)	0.18 (0.38)	0.009	--
Death (%)	21 (13.6%)	12 (14.5%)	11 (10.1%)	14 (19.4%)	30 (16.0%)	7 (10.9%)	0.46	18.8%
AUC-ROC (95CI%)	0.78 (0.67–0.89)	0.93 (0.86–0.99)	0.86 (0.76–0.95)	0.84 (0.75–0.92)	0.80 (0.70–0.87)	0.80 (0.73–0.96)	0.017^+^	0.87 (0.82–0.91)

Data are presented as mean ± SD. unless otherwise stated. AUC-ROC (Area under curve ROC) for all-cause mortality; *FEV1* forced expiratory volume in 1 s, *mMRC* Modified Medical Research Council, *PA Pseudomonas aeruginosa*

*between Latin America centers

^+^ *p* value between series from Argentina and Brazil 1

**Exacerbation data does not include hospitalizations

**Fig. 2:**
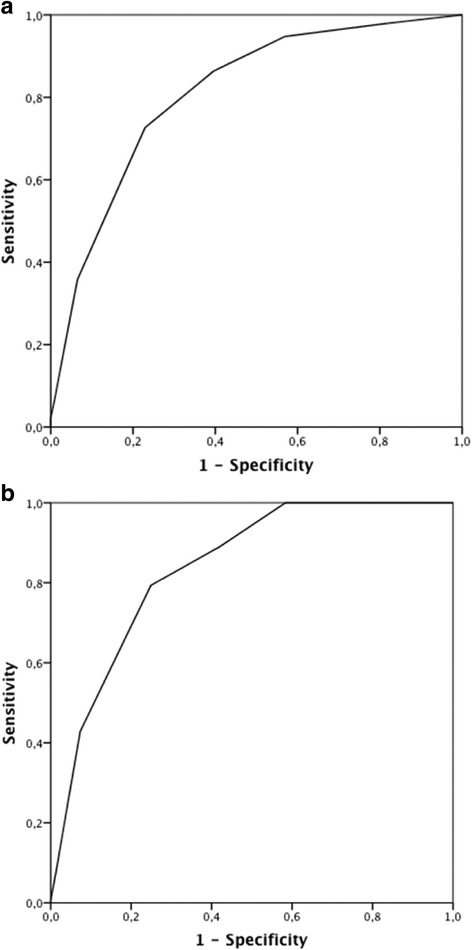
Receiver operating characteristic curves and area under the curve (AUC) to determine the overall predictive value of all-cause mortality (**a**) and respiratory mortality (**b**)

Figure 2a shows that the AUC ROC of the final score to predict 5-year all-cause mortality was 0.81 (95% CI: 0.77 to 0.85), *p* < 0.0001 and 0.84 (95% CI: 0.80 to 0.88), *p* < 0.0001 to predict respiratory mortality (Fig. 2b). The AUC ROC to predict all-cause mortality was calculated for each Latin American center and the observed values were higher than 0.80 in all except for one (AUC 0.78, range 0.78-0.93).

**Fig. 3:**
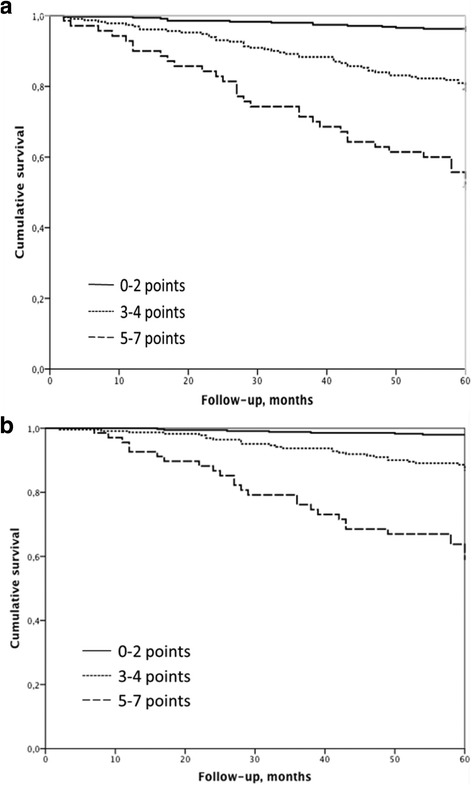
Kaplan-Meier curves for all-cause mortality (**a**) and respiratory mortality (**b**) corresponding to the three bronchiectasis scoring groups. Mild: 0–2 points, Moderate: 3–4 points and Severe 5–7 points. Log-rank test (**a**): mild bronchiectasis *versus* moderate bronchiectasis 43.29, *p* < 0.0001; mild *versus* severe 138.91; *p* < 0.0001; and moderate *versus* severe 23.42, *p* < 0.0001. Log-rank test (b): mild bronchiectasis *versus* moderate bronchiectasis 28.29, *p* < 0.0001; mild *versus* severe 127.51; *p* < 0.0001; and moderate *versus* severe 28.29, *p* < 0.0001

Figure 3a and b show how the division of the FACED score values into tertiles differentiated bronchiectasis into three distinct groups of increasing all-cause and respiratory mortality respectively according to the results of the Kaplan–Meier curves: mild (3.7% mortality), moderate (20.7% mortality) and severe (48.5% mortality) bronchiectasis.

Finally, to analyze the capacity of the FACED score to predict future exacerbations, we explored several scenarios based on the number and/or severity of exacerbation rates. Table 3 shows that the FACED score can predict those patients with more severe exacerbations (at least 1 hospitalization/year – AUC = 0.82 [95% CI: 0.78–0.87]) and patients with more frequent and relevant exacerbations (at least 2 exacerbations/year or 1 hospitalization/year – AUC = 0.78 [95% CI: 0.74–0.82]).

**Table 3:** Predictive value of FACED score on number and severity of future exacerbations and hospitalizations in the Latin America cohort

	AUC-ROC (FACED)	Best cut-off points (sensitivity/specificity)
At least 1 exacerbation/year (*n* = 228; 35.0%)	0.70 (0.67–0.75)	NA
At least 2 exacerbation/year (*n* = 117; 17.9%)	0.72 (0.68–0.78)	NA
At least 1 hospitalization/year (*n* = 56; 8.6%)	0.82 (0.78–0.87)	>3.5 points (Sensitivity: 79% and Specificity: 78%)
At least 2 exacerbation or 1 hospitalization/year (*n* = 150; 23.0%)	0.78 (0.74–0.82)	>2.5 points (Sensitivity: 82% and Specificity: 70%)

*NA* not applicable

## Discussion

In this study of Latin America validation of the FACED score, using the same methodology for data collection in six large historical cohorts of patients from three countries in Latin America with different clinical features and etiologies from the original cohort, the FACED score maintained an excellent power to predict all-cause and respiratory mortality. The FACED score also maintained its excellent discriminatory power by identifying a profile of increased severity in patients with bronchiectasis that was similar to that seen in the original Spanish study that constructed and internally validated this score. We also demonstrated that the score has a good capacity to predict exacerbations and hospitalizations, especially in those patients who present most severe or frequent exacerbations. These findings endorse the international clinical applicability of the FACED score as a prognostic and severity-assessment tool in patients with bronchiectasis.

There are still large gaps in our knowledge of the epidemiology and pathophysiology of bronchiectasis. The European Multicentre Bronchiectasis Audit and Research Collaboration (EMBARC) recently outlined several research priorities for improving the management of bronchiectasis [14, 15], including greater understanding of its classification and prognosis. Since there is a tendency to manage bronchiectasis based on approaches used for chronic obstructive pulmonary disease (COPD) and cystic fibrosis [16, 17], FEV_1_ has traditionally been used to define the severity of bronchiectasis. However, other key variables besides FEV_1_, such as age, quality of life, airway chronic colonization and lung volumes [18–21], have proved useful in this respect by showing a correlation with mortality in bronchiectasis. As many factors play an important role in the prognosis for bronchiectasis, just one of these on its own is obviously inadequate for predicting an outcome. For example, although CT findings are important and provide substantial information about structural abnormalities in airway diseases, CT scans are not sufficiently sensitive to detect the severity of functional impairment [22–24]. Furthermore, CT findings of airway diseases such as bronchial dilation or peribronchial wall thickening are not easily modifiable with treatment. However, decline in lung function also correlates with clinical parameters, the number and severity of exacerbations, and the presence of chronic colonization by *P. aeruginosa*, and it has been used to predict outcomes in bronchiectasis [18, 25]. Scores covering multiple variables that are validated in different populations of patients can therefore be key tools for assessing patients’ outcomes and their response to treatment. This scenario is even more complex when several comorbidities can coexist and negatively impact the prognosis of patients with bronchiectasis, as recently described by McDonnell et al [26]. Consequently, one of the prerequisites for the applicability of a multivariate score is confirmation of its external validity, i.e., its sustained ability to predict and diagnose regardless of the specific characteristics of the evaluated population. It is therefore essential to test such a score in settings different from the one in which it was first created and validated. One single-center study conducted in London by Ellis et al [9] in a small number of patients (but with a long follow-up of 19 years), and another study in Macedonia [27] both found that the FACED score had excellent predictive power. More recently, McDonnell et al [10] evaluated both the FACED and BSI scores in a large population comprising seven European cohorts and confirmed their discriminatory predictive value for mortality. However, it should be emphasized that all these studies used a European population base. Herein we present the first study to evaluate the FACED score in a group of patients with different ethnic characteristics in various Latin American countries since, as already demonstrated, ethnicity can influence several characteristics in bronchiectasis patients [28].

One of the most striking features of the FACED score is its simplicity. It is very easy to memorize by using the acronym and dichotomization of its variables (Fig. 1). This advantage increases its clinical applicability, making it useful even in the absence of digital equipment. The findings of this study confirm an excellent external validation of the FACED score for predicting both all-cause and respiratory mortality in six cohorts of patients with AUC-ROC above 0.8 from three Latin American countries. Thus, no significant deviations were found from the original prognostic power of the FACED score in the full cohort of 819 patients as regards general mortality (AUC-ROC 0.87 *versus* 0.81; *p* = 0.29) or respiratory mortality (AUC-ROC 0.85 *versus* 0.84; *p* = 0.88). The results were robust since the AUC-ROC range in the different countries was similar, and excellent in every case (range 0.78–0.93). One important aspect of this study is that the external validation was performed in patients with different characteristics from those of the original cohort. The patients included in the Latin American cohort were younger, with a greater number of affected lobes, and a higher prevalence of purulent sputum. Moreover, the Latin American patients presented worse lung function and a higher rate of chronic colonization by *P. aeruginosa*– all of which can interfere in bronchiectasis management [29, 30]. The external validation cohort also demonstrated a statistically significant discriminatory power with respect to the three degrees of severity (mild, moderate and severe) in patients with bronchiectasis. In this study, we were able to test an additional important use for the FACED score: its good power to predict exacerbations and hospitalizations (especially those patients with the most severe or frequent exacerbations), which are well recognized as surrogate markers of long-term unfavorable outcomes and potential target endpoints tor new treatments of bronchiectasis [31, 32].

The main limitation of this study is the fact that external validation in three Latin American countries does not guarantee the FACED score’s immediate applicability in countries with very different characteristics, such as Asian countries or the United States, where the frequency of tuberculosis etiology or non-tuberculosis mycobacteria is higher respectively [33, 34]. Validation is still required in other contexts to guarantee the worldwide applicability of the FACED score, although its validation in Latin America in patients with different clinical characteristics, as well as its validation in an English cohort with a long follow-up [9], suggests that it could also be applicable to other countries. A multidimensional score is a useful and easy-to-use tool for predicting and classifying the severity of patients, as well as for allowing comparisons between different cohorts and different treatments. The inclusion of other elements in the score that affect functional deterioration and worsen the quality of life in bronchiectasis, such as exacerbations or hospitalizations, could further strengthen the prognostic power of this instrument. However, it is essential to avoid any undue complication of the FACED score, since its simplicity is probably one of its main virtues and clearly guarantees its wide clinical applicability, especially in out-patient settings with no computers, as in the case of some Latin American centers [35]. In such situations, physicians need a usable tool that is easy to memorize, and the FACED score is ideal for this purpose. It should also be stated that, according to the nature of the retrospective studies, it is possible to occur biases in the collected information by not previous adequately completing of medical records. Such issue could have more impact in variables such as exacerbations, especially mild cases, and may be underestimated in the present study. For this reason, we prioritize hospitalization data for future risk assessment among patients with bronchiectasis in the present study.

## Conclusion

In conclusion, the FACED score maintained its excellent ability to predict all-cause and respiratory mortality when tested in a Latin American population. The score’s ability to discriminate between different degrees of bronchiectasis severity was also externally validated and proved similar to the findings in the original cohort. Furthermore, we significantly enhanced the clinical relevance of the FACED score since we also demonstrated its good discriminatory capacity for predicting multiple and severe exacerbations. Further research is also required to assess its ability to predict other important outcomes, such as quality of life or decline in pulmonary function.

## Additional file

Additional file 1: e-Appendix 1 – List of ethical committees and approval numbers. (DOCX 13 kb)

## Abbreviation

AUC-ROC: Area under ROC curve
BSI: Bronchiectasis Severity Index
CI: confidence interval
COPD: chronic obstructive pulmonary disease
CT: computed tomography
FEV1: forced expiratory volume in 1 s
FVC: forced vital capacity
mMRC: modified Medical Research Council
NA: not applicable
PPM: potentially pathogenic microorganisms
SD: standard deviation
