# Supplementary material for: Latin America validation of FACED score in patients with bronchiectasis: an analysis of six cohorts
Source: BMC Pulm Med. 2017 Apr 26;17:73. doi: 10.1186/s12890-017-0417-3 (PMC5406918; doi:10.1186/s12890-017-0417-3)
Supplement: Additional file 1: — e-Appendix 1 – List of ethical committees and approval numbers. (DOCX 13 kb) [file 12890_2017_417_MOESM1_ESM.docx]

e-Appendix 1 – List of ethical committees and approval numbers

| Center | Committee name | Approval number |
| --- | --- | --- |
| Heart Institute (InCor) do Hospital das Clinicas da Faculdade de Medicina da Universidade de São Paulo, São Paulo. Brazil | Cappesq | 1.267.294 |
| State University of Campinas (Unicamp),­ São Paulo. Brazil | COMITÊ DE ÉTICA EM PESQUISA DA UNICAMP - CAMPUS CAMPINAS | 1.142.117 |
| Hospital del Tórax. Dr A. Cetrángolo ­ Buenos Aires. Argentina | Sala de Docencia e Investigación | Vicente López, 20 de marzo de 2015 |
| Hospital Octávio de Freitas ­ Recife. Brazil | CAAE | 42747215.2.1001.5200 |
| Hospital de Messejana ­ Fortaleza. Brazil | CAAE | 42747215.2.1001.5200 |
| Instituto Nacional del Tórax ­ Santiago De Chile. Chile | CEC SSMO | 23082016 |
